# A Novel Approach in Sorting Chirality Species of Single-Wall Carbon Nanotubes Based on an Aqueous Two-Phase System of Polymer-Salt

**DOI:** 10.1038/s41598-020-58993-6

**Published:** 2020-02-06

**Authors:** Marziyeh Karandish, Somayeh Fardindoost, Gholamreza Pazuki

**Affiliations:** 1 Department of Chemical Engineering, Amirkabir University of Technology (Tehran Polytechnic), Tehran, Iran; 2 Department of Physics, Sharif University of Technology, Tehran, Iran

**Keywords:** Chemical engineering, Nanoscale materials

## Abstract

Sorting of distinct (n, m) chirality species of single-wall carbon nanotubes (SWCNTs) is essential for progress in technical applications in the field of electronic and optic devices. The purpose of this study is to investigate the isolation of single-wall carbon nanotubes based on diameters/chirality in a polymer-salt (polyethylene glycol and sodium citrate) aqueous two-phase system (ATPS) a substitute for common polymer-polymer (polyethylene glycol and dextran) system. The ATPS based on polymer-salt used instead of the common polymer-polymer system due to low viscosity, reduced surface tension, and lower cost of sodium citrate compared to the dextran. For this purpose, the ratio of concentrations of polyethylene glycol to sodium citrate as well as the effect of temperature on the isolation are both investigated and the selectivity and the recovery estimated approximately. The absorbance spectra from both top and bottom phases at different polymer and salt contents and at different temperatures show that by using this system in optimal conditions of polymer to salt ratio of 2:1 at temperature of 20 °C, a suitable separation of nanotubes with 85% yield of the chiral groups of 9 and 10 can be obtained.

## Introduction

Theoretically, SWCNTs as one of the carbon’s allotropes can be considered as graphene-like strip rolled up in a particular direction^1^. They have a one-dimensional structure with sp^2^ type bonding between adjacent carbon atoms^2–4^. Based on the rolling-up direction of the graphene layer and the angle of the nanotube’s hexagonal carbon-atom lattice, the chiral vector (n, m) is defined which determines many properties of SWCNTs especially their electrical and optical properties^2^. The variation in the chiral angle and their diameters cause a dramatic difference in their application in electronic and optoelectronic devices. So, electronically homogeneous SWCNTs are required for devices in field-effect transistors^5^, supercapacitors^6^, solar cell electrodes^7^, and biological sensors^8^. In the last decades, efforts have been made to structure-control of SWCNTs during growth process based on many methods mainly include arc discharge^9^, chemical vapor deposition (CVD)^10^, and the laser ablation^11^. Although the controlled growth of SWCNTs has made significant progress in the past decade, however, this process results in a mixture of different chirality of SWCNTs with low purity. Also, the low output of the chiral produced by these methods and the difficulty of step-by-step control of the production process, on the other hand, have attracted researchers’ attention to the post-synthesis process. So, many efforts have been made to isolate/sort the diameter/chirality of SWCNTs based on post-growth treatment methods^12^.

There are two basic strategies for post-synthesis treatment. In the first approach, the separation is based on the covalence bonding by functionalizing the wall of the nanotube. In this method, the inherent properties of SWCNTs are changed which affects their suitability for electrical applications. The second approach is based on solution-sorting to isolate non-covalently by dispersing the SWCNTs into a solution. In this method, the isolation is based on the selective physical adsorption of polymer or the surfactant on the SWCNTs which has demonstrated to have little effect on their electronic properties. Various methods have been proposed for this kind of separation among which the most important ones are density gradient centrifugation (DGU)^13^, chromatography^14^, selective dispersion with polymer^15^ and aqueous two-phase system (ATPS)^16^. These methods exhibit the mass structure separation of SWCNTs, which could dramatically accelerate its applications. However, high cost, long separation time, and limited life span of chromatography columns prevent them from commercialization. The aqueous two-phase separation (ATPS) was first introduced as a method of separation by Alberston in 1950 for the isolation of biomolecules and cell components^17^. In this method, two water-soluble components are mixed and reach a thermodynamic phase transition at a suitable concentration ratio to form two phases. The concentration of the constituents to reach the two-phase state depends on the chemical properties of each component and its molecular mass distribution. The binodal curve helps to determine the required concentration to form a two-phase system. The selective partitioning of the target material occurs when its components have different chemical affinities for distributing in the two phases. Afterward, the ATPS developed for the isolation of SWCNTs^18^. This method is based on the chirality isolation of the nanotubes in two phases with different amounts of hydrophobia ratios. It has been considered for its simplicity in commercialization and low cost since 2013 for the separation of SWCNTs^19^. The polyethylene glycol (PEG) and dextran (DX) are the most common two-phase systems used to isolate SWCNTs. The SWCNTs based on their chiralities are wrapped by surfactant micelles according to their different hydrophobicity and will be distributed between two phases. Among the advantages of this aqueous two-phase system, the high cost of dextran polymer, high viscosity and low access to the polymer can be pointed out as disadvantages.

The ATPS based on the polymer-salt systems previously have been introduced and developed for biomolecular separation based on different types of salts^20–23^. Among them, the polyethylene glycol-citrate salt system is one of the suitable aqueous two-phase systems to separate these target materials. On the other hand, the separation in the two-phase aqueous polymer-salt system is based on the salting out effect due to hydration or dehydration. It is also worth noting that the ion charge plays an important role in separation. The sodium citrate is a trivalent salt of the Hofmeister series and in this series, the trivalent foundations have more salting out effect than monolithic anions. According to the theory of exclusion, the effective excluded volume (EEV) is a parameter for comparing and estimating the ability of the salts to create salting out effect and to drive the more hydrophilic molecules. In other words, larger (EEV) is more likely to induce the salt outflow at a lower polymer concentration. On the other hand, our polyethylene glycol-citrate salt system has an (EEV) at the order of 64 which is the highest EEV compared to other reported salts^24^. Accordingly, sodium citrate (Na_3_C_6_H_5_O_7_) is a suitable choice for developing a two-phase system for SWCNTs separation. To the best of our knowledge, using salt as a constituent of ATPS for separation of CNT has not been investigated so far. Therefore, in this study the sodium citrate salt has been substituted for dextran polymer due to its degradability, high recovery and low cost^25^. We present the results of the chirality classification of SWCNTs using the ATPS method based on the PEG-sodium citrate salt system. The effect of the salt concentration investigated by making different ratios of PEG to salt. Also, the recovery of the optimum concentration was investigated at different temperatures. According to the work presented by Bruce Weisman and his colleagues, the chiral can be determined by having the wavelength of the absorption peaks^26,27^. Thus, the UV-visible-NIR absorbance spectrum was used to determine the chiral species. Finally, we calculated the efficiency and the selectivity of the samples and presented a separation mechanism with the help of FTIR spectroscopy.

## Materials and Methods

### Materials

SWCNTs of high purity with 60% outside diameter (OD) between 1–2 nm were purchased from Exir Company of Austria. For separating SWCNTs versus their (n, m) species, PEG (molecular weight (MW) = 6000 Da), and trisodium citrate dehydrate (Sigma Aldrich) were used as the main components of the two-phase system of polymer-salt. The sodium deoxycholate (DOC) 97% and sodium dodecyl sulfate (SDS) 98% from Sigma Aldrich were utilized as surfactants in this study.

### Methods

The SWCNTs (1 mg/ml) were initially dispersed in deionized (DI) water using 20 mg/ml DOC. Then the mixture was homogenized with the help of an ultrasonic bath for 1 hour. Next, the sonicated suspension was centrifuged at 9000 rpm for 30 minutes to remove the bundled SWCNTs. The purchased starting material of SWCNTs included variability in chirality distribution. The ATPS based on PEG-salt as a function of different PEG: salt concentration ratios at constant concentrations of surfactants investigated at PEG: salt ratios of 2:1, 1.5, 1 and 0.6 under the name of F1, F2, F3, and F4 respectively. These four systems with different concentration of components in each phase are selected based on the binodal diagram of polyethylene glycol- citrate salt in the two-phase region presented in Table S1 and Fig. S1. As shown, the concentration ratio of 2:1 is close to the binodal curve and has the shortest tie line. On the other hand, the ratio of 0.6 is the farthest point from the binodal curve. Samples F1 with PEG: salt ratio of 2:1, was prepared containing: 1.43 g PEG, 0.714 g trisodium citrate dehydrate, 0.7 g of SDS 10%, 0.7 g of DOC 10%, and 3.6 g deionized water. It was mixed for 30 minutes and then the prepared suspension of SWCNTs (0.5 ml) was added to this solution followed by mixing by vortex for 30 s. The mixture was put in an incubator for 20 minutes and then centrifuged for 2 minutes at 3000 rpm in order to accelerate phase separation. This procedure was repeated for three other samples (F2-F4).

The absorption spectroscopy with a high-throughput characterization of SWCNTs based on their diameters was used to investigate the separated (n, m) species in each phase of the system. The absorption spectra were recorded from 900–1300 nm using Perkin Elmer (lambda 950) UV-VIS spectrophotometer for SWCNTs solution (starting material), the top and bottom phases of ATPS after chiral isolation. According to the obtained recovery results from the absorption spectra, sample F1 with PEG: salt ratio of 2:1 was investigated at different temperatures of 10, 20, 25 and 30 °C.

## Results and Discussions

### Chiral identification

**Figure 1:**
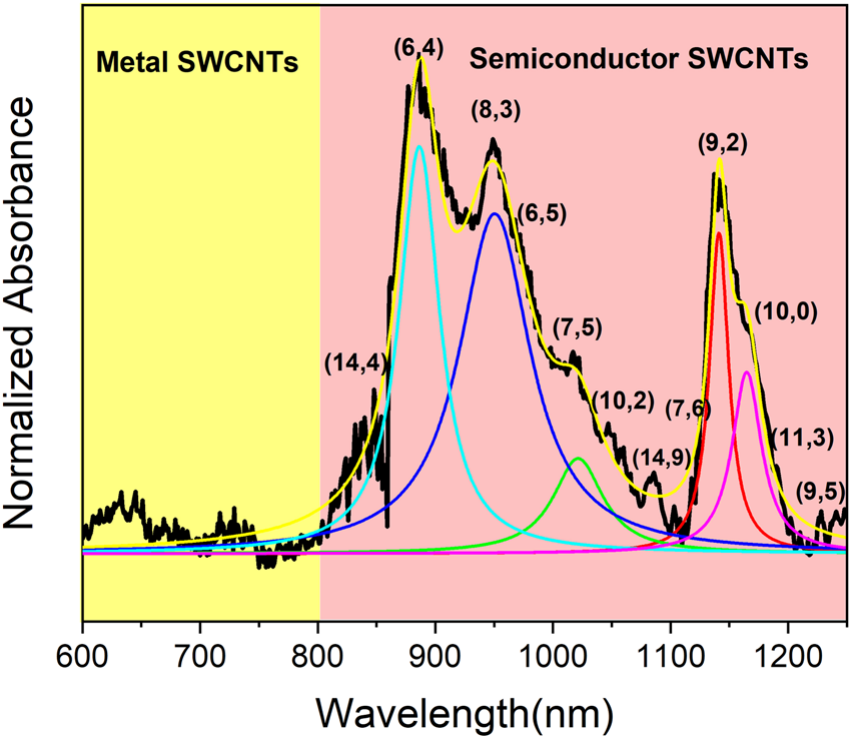
Absorption spectrum of the SWCNTs solution as a starting material, the first optical transition (E11) and the second (E22) show metal and semiconductor SWCNTs respectively.

The chiral composition was analyzed carefully by UV-visible-NIR absorbance spectra collected in a 1 nm increment through a quartz cuvette. During the analysis, the corresponding blank surfactant/polymer solution spectra were linearly subtracted. Each SWCNT species can absorb light at a distinct wavelength dedicated by their chirality (n, m). The electronic states’ density of SWCNTs includes sharp van Hove peaks and the optical transition between these peaks with specific energies corresponds to a specific chiral (n, m) in absorption spectroscopy^26,27^. The purchased SWCNTs have polydispersity in diameter/chirality, so firstly we studied the variability in chirality distribution of the starting material. Figure 1 shows the absorption spectra of the SWCNTs dispersed in DI water and DOC. The first and second optical transition (E11) and (E22) are indicated by the shaded region in different colors. As can be seen, there is no particular absorption peak below 800 nm (the yellow part, E11 region), which is related to metal transmissions. Most of the absorption peaks range from 800 to 1300 nm (the pink region (E22)) related to semiconductor transmissions. So, it can be concluded that most of the nanotubes are semiconductors with diameters of more than 0.7 nm. Due to the close distribution in the diameter’s size and the SWCNTs agglomeration, the obtained UV-VIS absorption spectrum of SWCNTs solution (the starting material) includes the broadening and overlapping peaks, which are clearly identified in Fig. 1. Also, the wavelengths correspond to each absorption peaks are in accordance with the diameter distribution of 0.7 to 2 nm, predominantly consist of the chiral of (6,4), (10,2), (9,2), (9,5), (8,3), and (10,0).

### Concentration effect

The distribution of SWCNTs in the two phases of PEG and sodium citrate determined with the help of a binodal curve (Table S1 and Fig. S1). The concentrations of the components in each phase determined by simultaneously solving the following four Eqs. (1–4) according to the work of Marchuk and his colleagues by assuming that it is a three-component system as well as ignoring the small amount of surfactants^28^.

1 $${Y}_{T}={\rm{Aexp}}\{(B{X}_{T}^{0.5})-(C{X}_{T}^{3})\}$$

2 $${Y}_{B}={\rm{Aexp}}\{(B{X}_{B}^{0.5})-(C{X}_{B}^{3})\}$$

3 $${Y}_{T}=\frac{{Y}_{M}}{{\rm{\alpha }}}-\frac{1-{\rm{\alpha }}}{{\rm{\alpha }}}{Y}_{B}$$

4 $${X}_{T}=\frac{{X}_{M}}{{\rm{\alpha }}}-\frac{1-{\rm{\alpha }}}{{\rm{\alpha }}}{X}_{B}$$

Equations (1) and (2) are obtained empirically from binodal curve, and Eqs. 3 and 4 are derived from the mass balance of the two-phase system. In these equations, Y is the weight fraction of polymer, X is the weight fraction of salt, M indicates the initial mixture, top phase (T), bottom phase (B), and the parameter α represents the weight ratio of the T to B. According to the Table 1, from 1 to 4 feeds (F1-F4), the weight percentage of PEG ranged from 41 to 52%, and the weight percentage of salt ranged from 3.5 to 2.5%. Also, in the B phase, the weight percentage of PEG ranged from 6% to about 1%, and for salt ranged from 14% to 42%.

**Table 1:** The components’ weight percentage in feed mixture and in each phases of top and bottom.

Test number	Mixture composition (wt%)		Top phase composition (wt%)		Bottom phase composition (wt%)	
	Salt	PEG	Salt	PEG	Salt	PEG
F_1_	10	20	3.35	41.50	14.53	6.82
F_2_	14	20	2.92	46.46	19.18	3.80
F_3_	20	20	2.62	50.55	30.65	1.58
F_4_	30	20	2.53	52.02	42.84	0.86

**Figure 2:**
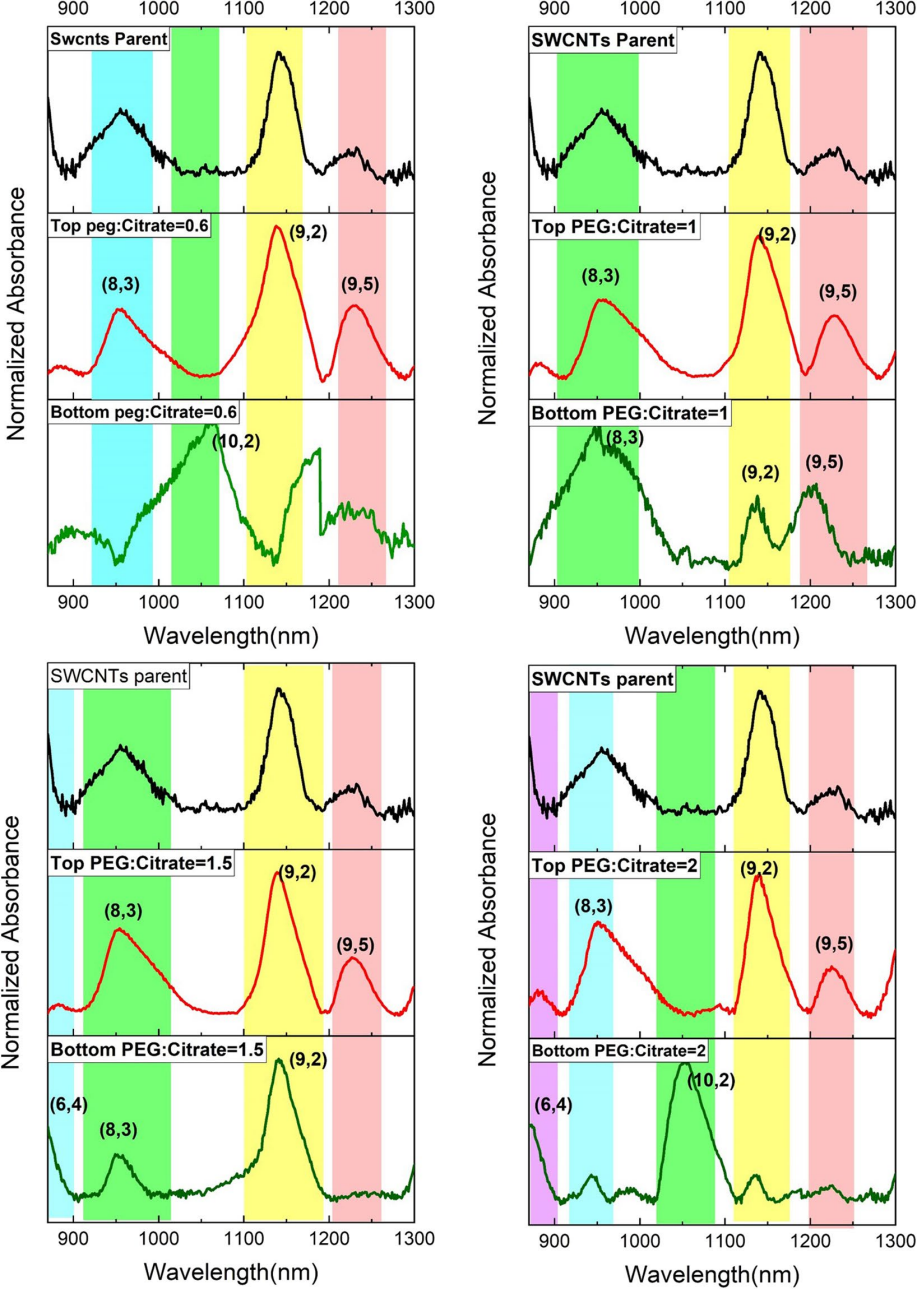
Absorption spectra of the separated chiralities of SWCNTs in the top and bottom phases at different PEG: salt ratios of 2, 1.5, 1 and 0.6.

Secondly, the chiral isolation investigated in a PEG-salt system as a function of PEG: salt concentration ratios of 2, 1.5, 1 and 0.6 at a constant concentration of surfactant. The results of absorption spectra are shown in Fig. 2 for F1 to F4. According to the 12 chiralities in the starting material shown in Fig. 1, total of five chiralities are sorted in the top and bottom phases and the rest are lost at the interface. As can be seen from the absorption spectra in Fig. 2, in all samples the top phase consists of a selected set of three chiralities of (9,2), (9,5), and (8,3) as highlighted by the shaded region in colors. But in the bottom phase, the distribution of the separated chiralities vary by the concentration of citrate salt. Increasing the concentration of the citrate salt causes the broadening in the absorption peaks in the bottom phase, indicating that there is an agglomeration in the system. However, the system of PEG: citrate salt with ratio 2:1 (F1), isolate selectable the chiral (10, 2) and (6, 4) at the bottom phase which was retained in the other systems (F2-F4) at the interface.

### Temperature effect

**Figure 3:**
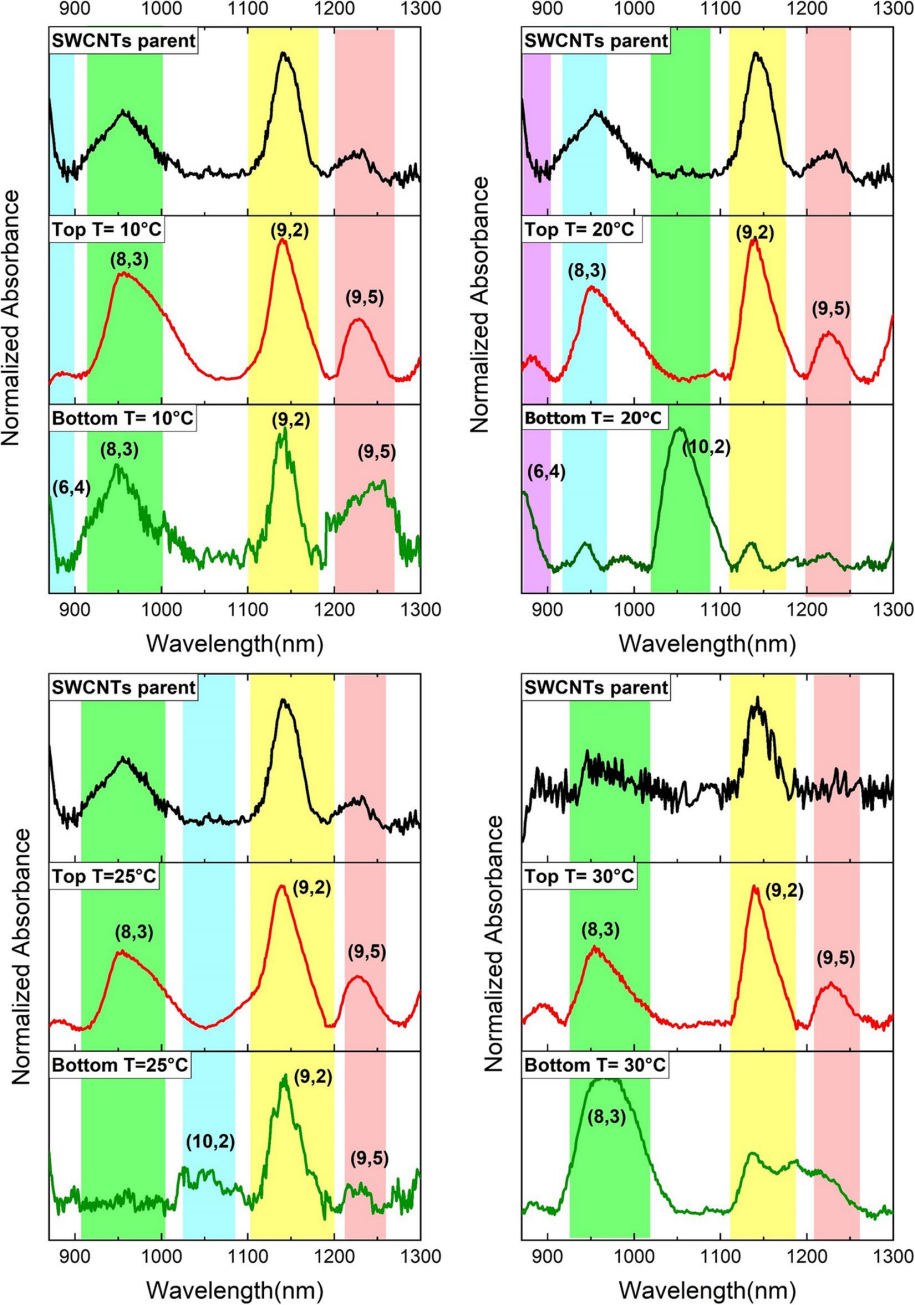
Absorption spectra of the separated chiralities of SWCNTs in the top and bottom phases of PEG: salt 2:1 at temperatures of 10, 20, 25, and 30 °C.

The temperature has a direct effect on solubility and viscosity and it is one of the most effective parameters for the separation. So, thirdly, the separation in a PEG-salt system as a function of temperature at 10, 20, 25 and 30 °C investigated for PEG: salt ratio of 2 (F1) which was the most selectable system among F1-F4. We used an incubator with a temperature range of minus 5 to 70 °C to apply and control the temperature media. So, after adding SWCNT to the two-phase system, they were incubated for 45 min at the required temperature. The resulting absorption spectra are displayed in Fig. 3. As observed for all the temperatures, the top phase consists of the same chiralities and considerably the temperature effects on the selective chiral sorting in the bottom phase. At 10 °C, the chiral distribution in the bottom phase is similar to the starting material of SWCNT (Fig. 1), which can be attributed to the decrease in the surface energy of the nanotubes at a low temperature of 10 °C. By increasing the temperature from 20 °C to 25 °C and then to 30 °C, the absorption peaks at bottom phase start broadening. It can be attributed to desorption of the surfactants from the SWCNTs’ surface by temperature increment which results in SWCNTs agglomeration. In addition, increasing the temperature reduces the interaction between water and polymer molecules followed by transferring water molecules to the bottom phase. This results in a higher concentration of the polymer in the top phase and a diluted bottom phase, which also causes agglomeration and peak broadening^29,30^. Therefore, as evaluated, 20 °C is the optimum temperature for better sorting of SWCNTs based on their chiralities as obtained and displayed in Fig. 2.

The corresponding absorbance intensity of isolated chiral of (10, 2) and (9, 2) versus different PEG to salt concentrations and also at different temperatures for the system of PEG: citrate salt with ratio 2 (F1) after 5 times replication are shown in the Fig. S2. Also, the resonance Raman spectroscopy (RBM mode-diameter relation) taken from starting material, top and the bottom phase’ composition of F1 shown in Fig. S3. The assignment of each particular chiral to the RBM frequency is extracted from Katura plots of Eii transition energies vs tube diameters according to^27^. As shown in Fig. S3, we found the starting material includes the chiralities of (10, 9), (10, 2), (9, 2), (9, 5), (8, 3) with diameters below 2 nm. After the isolation, the top phase mostly includes (9, 2) and the (10, 2) distributed at the bottom phase which all are in accordance with the absorption spectrum (Fig. 2).

### Recovery

The chiral composition of each phase which analyzed by absorption spectra utilized to obtain the selectivity of the introduced system by calculating the recovery (*R*_*T*_) for each sample (Eq. (5)). *R*_*T*_ provides an overview of the chirality distribution into two phases. We balanced the mass between the top and bottom phases regardless of the mass in the interface^22^. In addition, the volumes of each phase *V*_*R*_ and the coefficient of distribution of SWCNTs (*K*) are calculated according to the equations of (6) and (7), respectively. In these equations, K is the ratio of the concentration of each chiral in the top phase to its concentration at the bottom phase.

5 $${R}_{T}=\frac{100}{1+\frac{1}{{V}_{R}K}}$$

6 $${V}_{R}=\frac{{V}_{TOP}}{{V}_{BOTTOM}}$$

7 $$k=\frac{{C}_{TOP}}{{C}_{BOTTOM}}$$

The absorption intensity is directly related to the concentration of the species. So, the concentration of each chiral in each phase obtained according to Beer Lambert’s relation, Eq. (8)^31^.

8 $${\rm{A}}={\rm{\varepsilon }}\text{bC}$$

*A* indicates absorbance, ε is the molar adsorption (*L mol*^−1^*cm*^−1^), *b* is the sample path length, and *C* is the concentration of the component in the solution, respectively. Therefore, K can be re-defined as the ratio of the absorption of each chiral in the top and bottom phases based on the following Eq. (9):

9 $${\rm{K}}=\frac{{A}_{TOP}}{{A}_{BOTTOM}}$$

10 $${s}_{12}=\frac{{K}_{1}}{{K}_{2}}$$

The selectivity for a system with two specific chiral with dominant concentrations is defined from the Eq. (10), where *s*_12_ is the selectivity of chiral 1 from chiral 2. The more *s*_12_ goes further than 1, the more selectable and suitable is the system for the separation of the two chiral. In this work, the partition coefficient, recovery, selectivity, and volume ratio *V*_*R*_ for each PEG:salt concentrations, as well as different temperatures of 20, 25 and 30 °C are reported in Tables 2 and 3, respectively. The presented results support a strong chiral sorting by considering the chirality distribution in our starting SWCNTs material. As shown in Table 2, from the total of 12 identified chiralities in the starting material as shown in Fig. 1, three chiralities isolated at the top phase and 2 chiralities remained at the bottom phase and the rest are lost at the interface. Approximately over 80% of the (9, 2), (8, 3), and (9, 5) can be found in the top phase and about 15% of the (10, 2) can be found in the bottom phase. Also by comparing F1 and F2, an increase in the concentration of polymer at the top-phase resulted in an increase in the recovery for three chiral. In F3 and F4, although both the concentration of polymer and the K increase but the recovery is reduced due to the low available volume (*V*_*R*_). Therefore, it can be concluded that an optimum concentration of polymer needs to reach to the highest degree of separation. Although the separation of chiral at the top phase in F2 is slightly higher, F1 operates more selective in the bottom phase for two specific chiral. Due to the agglomeration in the bottom phase for F3 and F4, no selectable chiral sorting is presented in Table 2.

**Table 2:** Partition coefficient, recovery, selectivity, and volume ratio in the top and bottom phases of F1, F2, F3, and F4. The selectivity for the top phases of F1 and F2 is calculated against (10, 2) and (6, 4) respectively.

Test number	Phase	Chirality	K	R_T_	Selectivity	V_R_
F_1_	Top	(9,5)	6.36	91.06	58	
		(9,2)	5.83	90.31	53	
		(8,3)	5.16	89.16	47	1.6
	Bottom	(10,2)	0.11	15.36		
		(6,4)	0.22	25.79		
F_2_	Top	(9,5)	8.85	93.02	68	
		(9,2)	2.98	81.70	22	1.5
		(8,3)	5.92	89.89	46	
	Bottom	(6,4)	0.13	16.83		
F_3_	Top	(9,5)	11.07	90.69		
		(9,2)	10.87	94.69	—	0.9
		(8,3)	4.39	86.82		
F_4_	Top	(9,5)	14.07	90.24		
		(9,2)	14.28	90.40	—	0.6
		(8,3)	6.92	82.07

**Table 3:** Partition coefficient, recovery, selectivity, and volume ratio in the top and bottom phases of F1 at different temperature. The selectivity for the top phases of F1 is calculated against (10, 2).

Temperature	Phase	Chirality	K	R_T_	Selectivity
20 °C	Top	(9,5)	6.36	91.06	53
		(9,2)	5.83	90.31	49
		(8,3)	5.16	89.16	43
	Bottom	(10,2)	0.11	15.36	
		(6,4)	0.22	25.79	
25 °C	Top	(9,5)	6.60	91.35	15.7
		(9,2)	6.58	91.42	15.6
		(8,3)	6.19	90.82	4.7
	Bottom	(10,2)	0.42	—	
30 °C	Top	(9,5)	4.32	87.36	
		(9,2)	4.01	86.53	—
	Bottom	(8,3)	0.6	49.00	

According to Table 3, K and *R*_*T*_ are also increased up to 25 °C but decrease at once at 30 °C, which can be due to a decrease in *V*_*R*_ due to the temperature rise or desorption of the active surfactants from the SWCNTs’ surface.

We repeated the steps of the separation for F1, but we observed that a significant fraction of the SWCNTs lost at the interface thus we did not obtain any considerable sorting by repeating the steps further and limited the number of consecutive separation steps to one.

### The separation mechanism

A detailed analysis of the separation mechanism is a comprehensive work that should be taken as the next step. Previous reports show that DOC as a surfactant forms a homogeneous micelle structure around the SWCNTs resulting in better dispersion and increase their solubility and hydrophilicity. On the other hand, SDS forms a disordered micelle structure which in the presence of the salts reforms to a more ordered surface structure^32^. So, DOC covered SWCNTs distribute into a more hydrophilic phase and SDS wrapped ones separate into a less hydrophilic phase. In this work the ratio of SDS: DOC is kept fix and the competition between both surfactants is manipulated by the presence of salt with different concentrations. The presence of sodium citrate in the aqueous two-phase system results in the formation of mixed salt-DOC micelles for the smaller diameters and mixed salt-DOC-SDS micelles for the larger diameters, redistributing them into the less hydrophilic phase. As presented in Fig. 2, the incremental addition of the salt results in the broadening of the absorption peaks at the bottom phase. By an increase in the salt concentration, we expect that the SDS restructures and cover around the SWCNTs more competitively compared to the DOC micelle structure, thus causes the agglomeration of smaller ones at the bottom phase.

**Figure 4:**
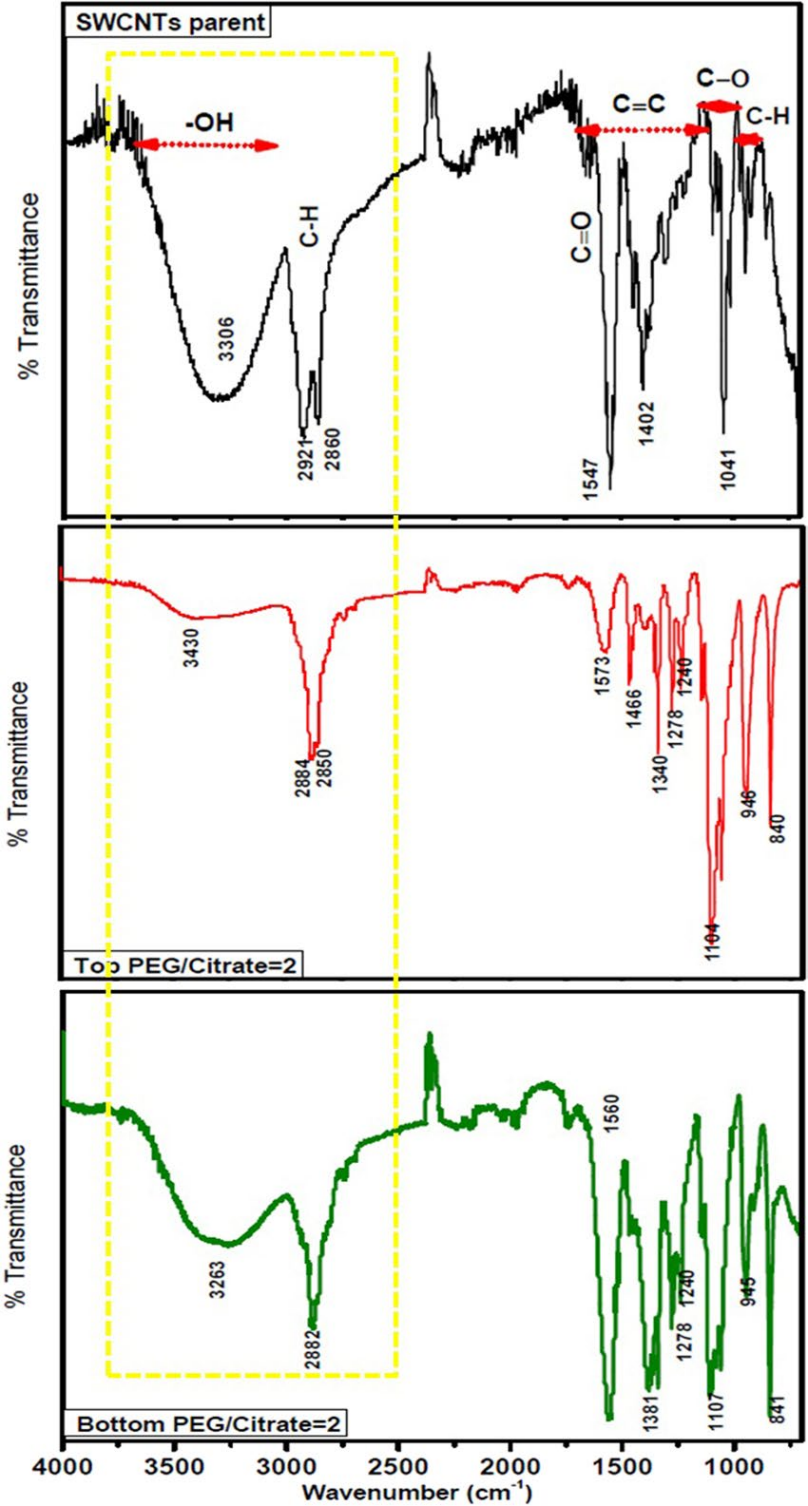
FTIR spectra of pristine SWCNTs, after separation at the top and bottom phase for F1.

We devoted special attention to the behaviour of the functional groups attached on the surface of the SWCNTs in sample F1 utilizing FTIR spectra in the wavenumber region 500–4000 cm^−1^. Figure 4 demonstrates the spectra of a comparative FTIR data for the pristine SWCNTs and after separation in the top and bottom phases. As observed in the pristine sample, the extremely wide and non-homogeneously broadened band stretching line of OH-groups apparently seen in the region 3200–3600 cm^−1^. Also, quite intense peaks of 2921 cm^−1^, 2860 cm^−1^ and the region of 1400 to 1600 cm^−1^ are corresponded to C–H stretch and aromatic C=C stretching bands groups respectively. The peaks at ∼2,921 cm^−1^ can be attributed to the protonation of the CNTs as a result of their interactions with DI water. Signals in the area of wave number 1000–1300 cm^−1^ can be attributed to the C-O linkages. After the separation, a considerable change and shifts in the signals observed in the region of 2800–3600 cm^−1^. In the top phase, due to the interactions between CNT-OH and hydroxyl-terminated PEG, an ester is formed whose carbonyl vibration may be easily assigned at the region of 1500–1700 cm^−1^ ^33^. The strong symmetrical and asymmetrical stretching of –CH2– bands at 2921 cm^−1^ and 2860 cm^−1^of pristine SWCNTs are shifted to 2884 cm^−1^ and 2850 cm^−1^ in the top phase and 2882 cm^−1^ in the bottom phase indicating the interfacial interaction of CH with the SDS, DOC and sodium citrate^34^. As clearly observed the top phase with less OH stretching band is less hydrophilic and the bottom phase with a strong broaden band of OH is more hydrophilic. According to this result, the SWCNTs with smaller diameters distribute at the bottom phase with more hydrophilicity.

**Figure 5:**
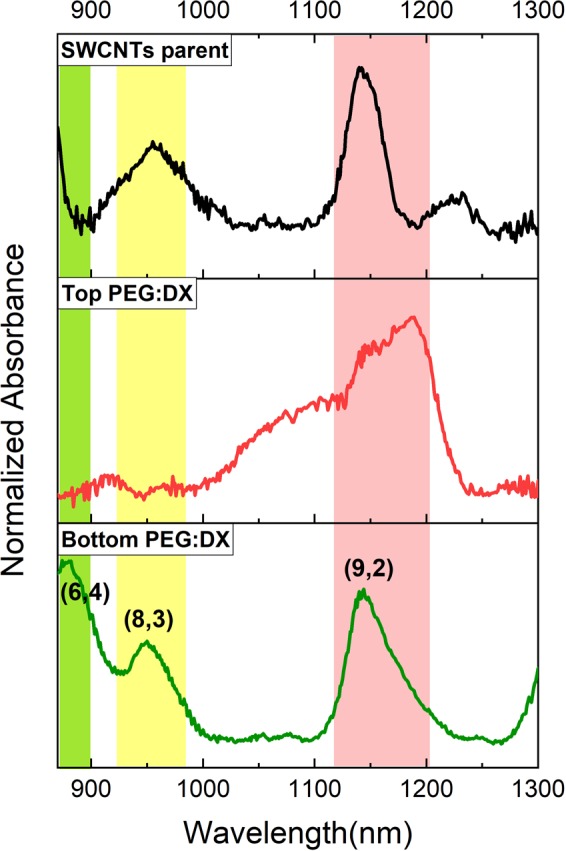
Absorption spectra of the separated chiralities of SWCNTs in the top and bottom phases of PEG: DX.

The chirality sorting of the introduced system compared with the common aqueous two-phase system of polyethylene glycol-dextran (PEG: DX). The sample of the two-phase system of PEG: DX was prepared according to the work of Wei *et al*.^35^. The results of the absorption spectroscopy are presented in Fig. 5. In the aqueous two-phase system of PEG: DX, selective separation of three-chiral (9, 2), (8, 3), and (6, 4) obtained in the bottom phase, but the peaks broadening was observed in the top phase. The interesting point is that in the PEG: DX system, the chiral (8, 3) isolated at the bottom phase which tends to be at top phase in the PEG: salt system. It indicates the difference of surface interactions of SWCNTs in these two systems. As reported by Fagan *et al*.^16^, in the ATPS separation based on PEG: DX, the PEG-rich phase contains more water compare to dextran-rich phase which makes it more hydrophilic. So, the chiral (8, 3) with less hydrophilicity is sorted at the bottom phase.

## Conclusions

We have demonstrated a novel approach in sorting chirality species of single-wall carbon nanotubes based on an aqueous two-phase system of PEG-sodium-citrate. The performance of this system evaluated studying the parameters of the concentration and the temperature effect. We have determined that the presence of sodium citrate as a salt in the aqueous two-phase system results in the formation of mixed salt-DOC micelles for the smaller diameters at the top phase and mixed salt-DOC-SDS micelles for the larger diameters, redistributing them into the less hydrophilic phase at the bottom phase. Isolation of specific chiralities in the top and bottom phases accomplished in a single step. The ATPS based on PEG: sodium citrate with a concentration ratio of 2 at 20 °C showed isolating the chiral families of 9 and 10 with 85% recovery. It was observed that temperature has no optimal effect on separation at the top phase but isolate selectively the (10, 2) at the bottom phase. With the replacement of citrate salt instead of dextran polymer, the cost of separation is greatly reduced. It can be said that development of ATPS based on the salt is an effective step towards industrialization of this method for SWCNTs chirality separation.

## Supplementary information

Supporting Information.
