# Supplementary material for: A Novel Approach in Sorting Chirality Species of Single-Wall Carbon Nanotubes Based on an Aqueous Two-Phase System of Polymer-Salt
Source: Sci Rep. 2020 Feb 6;10:2025. doi: 10.1038/s41598-020-58993-6 (PMC7005278; doi:10.1038/s41598-020-58993-6)
Supplement: Supplementary file 1 — Supporting Information. [file 41598_2020_58993_MOESM1_ESM.docx]

**Supplementary Information**

**A Novel Approach in Sorting Chirality Species of Single-Wall Carbon Nanotubes Based on an Aqueous Two-Phase System of Polymer-Salt**

Marziyeh Karandish ^a^, Somayeh Fardindoost ^b^ and Gholamreza Pazuki *^c^

*^a^ Department of Chemical Engineering, Amirkabir University of Technology (Tehran polytechnic), Tehran, Iran*

*^b^ Department of Physics, Sharif University of Technology, Tehran, Iran*

*^*c^ Department of Chemical Engineering, Amirkabir University of Technology (Tehran polytechnic), Tehran, Iran*

The binodal curve has been well adapted with the three-parameter Marchuk model (Equation 1), which is proposed for the determination of binodal curve of citrate aqueous two-phase system based on the experimental data. The parameters$A$, $B$ and $C$ in the Marchuk equation as well as the correlation coefficient parameter ($R^{\mathbf{2}}$) are all reported in table S2as indicators for assessing the conformity of the experimental data with the Marchuk equation. It should be noted that the correlation coefficient is calculated from equation 2, in which, n is the number of empirical points obtained for the formation of binodal curve. The Y_exp_, weight percent of the polymer obtained experimentally and Y_model_ is the weight percent of the polymer related to the calculated data by the Marchuk equation:

$\mathbf{Y}_{\mathbf{T}}\mathbf{=}\boldsymbol{Aexp\{}\left( \mathbf{BX}_{\mathbf{T}}^{\mathbf{0.5}}\mathbf{)-(}\mathbf{CX}_{\mathbf{T}}^{\mathbf{3}} \right)\boldsymbol{\}}$ (1)

$\mathbf{R}^{\mathbf{2}}\mathbf{=1-}\frac{\sum_{\mathbf{1}}^{\mathbf{n}} {\mathbf{(}\mathbf{Y}_{\mathbf{exp}}\mathbf{-}\mathbf{Y}_{\mathbf{model}}\mathbf{)}}^{\mathbf{2}}}{\sum_{\mathbf{1}}^{\mathbf{n}} {\mathbf{(}\mathbf{Y}_{\mathbf{exp}}\mathbf{-}\bar{\mathbf{Y)}}}^{\mathbf{2}}}$ $\bar{\mathbf{Y}}\mathbf{=}\frac{\sum_{\mathbf{1}}^{\mathbf{n}} \mathbf{Y}_{\mathbf{exp}}}{\mathbf{n}}$ (2)

TableS1 shows the binodal curve data for the polyethylene glycol-sodium citrate aqueous two phase system. These curves are obtained from the cloudy method at 20 ° C.

Table S1$\boldsymbol{:}$ Binodal curve data in weight percent for each component

| PEG6000+ Tri sodium citrate | | | | PEG6000+ Tri sodium citrate | | | | |
| --- | --- | --- | --- | --- | --- | --- | --- | --- |
| Water | Salt | Polymer |  | | Water | Salt | Polymer |  |
| 78.66 | 10.84 | 10.50 | 21 | | 48.49 | 2.75 | 48.75 | 1 |
| 78.74 | 11.03 | 10.21 | 22 | | 51.28 | 2.98 | 45.73 | 2 |
| 78.82 | 11.22 | 9.95 | 23 | | 54.28 | 3.26 | 42.45 | 3 |
| 78.86 | 11.34 | 9.81 | 24 | | 62.36 | 4.25 | 33.38 | 4 |
| 78.90 | 11.45 | 9.64 | 25 | | 70.13 | 5.81 | 24.05 | 5 |
| 78.93 | 11.54 | 9.52 | 26 | | 71.57 | 6.23 | 22.19 | 6 |
| 78.95 | 11.6 | 9.44 | 27 | | 72.70 | 6.61 | 20.68 | 7 |
| 78.97 | 11.68 | 9.33 | 28 | | 75.29 | 7.74 | 16.96 | 8 |
| 78.99 | 11.71 | 9.30 | 29 | | 75.80 | 8.03 | 16.16 | 9 |
| 79.00 | 11.82 | 9.21 | 30 | | 76.40 | 8.41 | 15.18 | 10 |
| 79.05 | 11.95 | 9.00 | 31 | | 76.89 | 8.77 | 14.34 | 11 |
| 79.07 | 12.04 | 8.89 | 32 | | 77.22 | 9.04 | 13.73 | 12 |
| 79.12 | 13.94 | 6.94 | 33 | | 77.74 | 9.55 | 12.70 | 13 |
| 78.89 | 15.09 | 6.04 | 34 | | 78.10 | 9.97 | 11.92 | 14 |
| 78.42 | 16.44 | 5.13 | 35 | | 78.14 | 10.02 | 11.83 | 15 |
| 78.13 | 17.12 | 4.74 | 36 | | 78.29 | 10.23 | 11.47 | 16 |
| 77.58 | 18.21 | 4.21 | 37 | | 78.33 | 10.29 | 11.38 | 17 |
| 77.03 | 19.19 | 3.77 | 38 | | 78.53 | 10.61 | 10.85 | 18 |
| 76.46 | 20.14 | 3.43 | 39 | | 78.60 | 10.74 | 10.65 | 19 |
| 75.49 | 21.55 | 2.95 | 40 | | 78.64 | 10.82 | 10.53 | 20 |

Figure S1 $\mathrm{pr}$esents the binodal curve for our aqueous two-phase system in the vertical angular coordinate.

Figure S1: Binodal curve of polyethylene glycol-trisodium citrate aqueous two-phase system in vertical angle coordinates

Table S2$\boldsymbol{:}$Constants of the Marchuk equation and correlation coefficient for aqueous two phase system

| $\boldsymbol{R}^{\boldsymbol{2}}$ | | $\boldsymbol{C}$ | | $\boldsymbol{B}$ | | $\boldsymbol{A}$ | | ATPS | |
| --- | --- | --- | --- | --- | --- | --- | --- | --- | --- |
| 0.9941 | 0.00012 | | -9.3933 | | 2.3147 | | trisodium citrate+polyethylene glycol+water | |  |

The corresponding absorbance intensity versus concentration ratio and temperature for chirality distribution of the system of PEG: citrate salt with ratio 2 (F1) after 5 times replication are shown in figure S2. As obtained from the absorption spectra in figs. 2 and 3, the chiral (10, 2) isolated selectable at 20 °C at the bottom phase and (9, 2) isolated at the top phase regardless of the concentration ratio and the temperature effect.


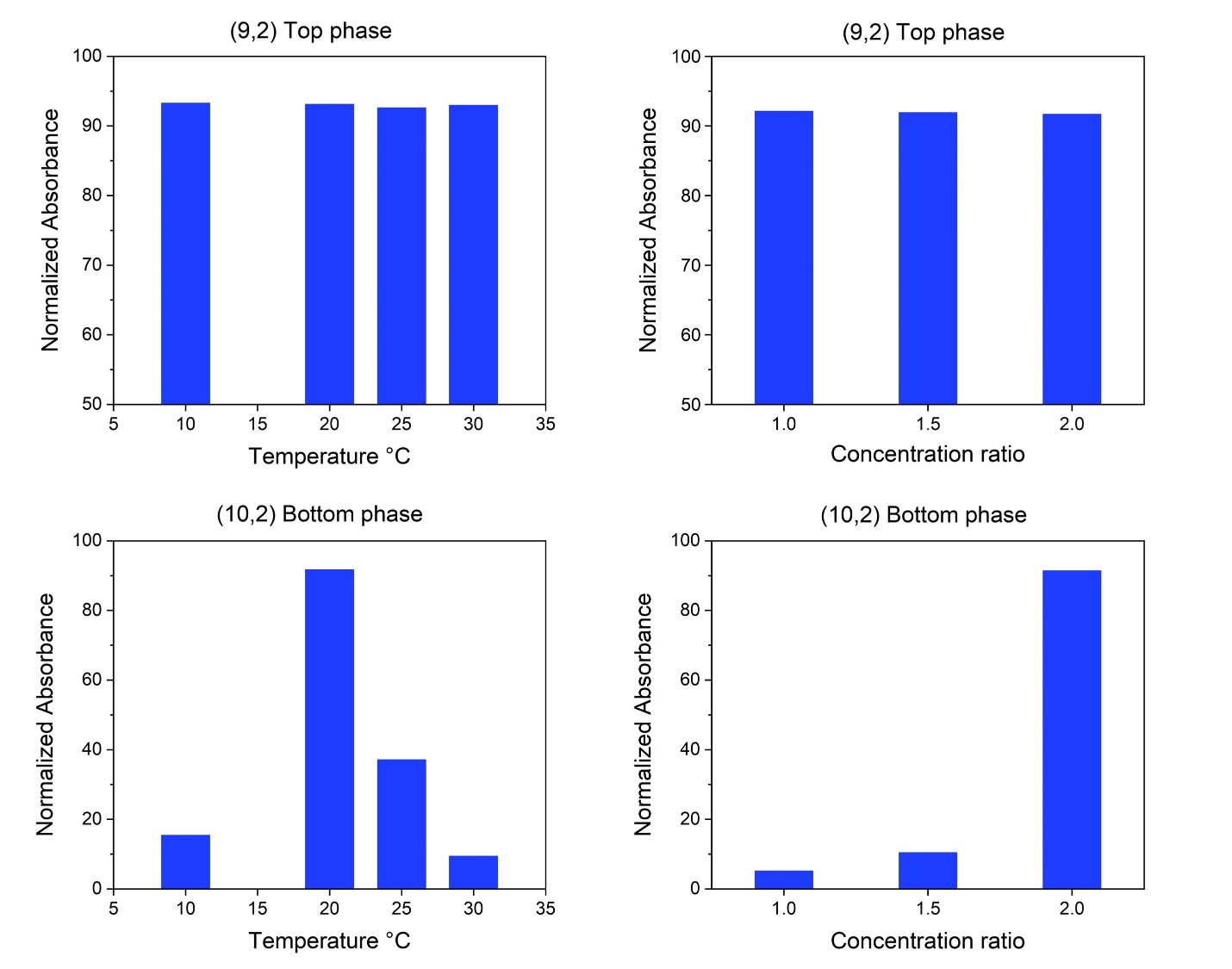


Figure S2. The corresponding absorbance intensity versus concentration ratio and temperature for chirality distribution of the system of PEG: citrate salt with ratio 2 (F1).

For the system of PEG: citrate salt with ratio 2 (F1), the resonance Raman spectroscopy (RBM mode-diameter relation) taken from starting material, top phase and bottom phase composition shown in figure S3. The radial breathing mode is the characteristic phonon mode of single-walled carbon nanotubes in which the atoms vibrate in-phase in the radial direction. The frequency of the radial vibration is linearly related to the inverse of the tube diameter. The Raman experiment requires various lasers for an assignment of (n, m) and at fixed laser radiation, some chiralities poses more intense peaks compared with others [27]. Also, the tubes environment affects phonons and optical transition energies of carbon nanotubes in Raman scattering. Accordingly, we observed in our results that the interaction between the tubes bundle can shift the optical transition energies to the red and lead to peak broadening in the RBM frequencies. The assignment of each particular chiral to a RBM frequency is extracted from Katura plots of Eii transition energies vs tube diameters according to [27]. As shown in figure S3 and compare to the absorption spectroscopy results, we found chiral distribution in two phases based on their diameters. The starting material includes the chiralities of (10, 9), (10, 2), (9, 2), (9, 5), (8, 3) with diameters below 2 nm. After the isolation, the top phase mostly includes the (9, 2) and the (10, 2) distributed at the bottom phase which is in accordance with the obtained absorption spectra.


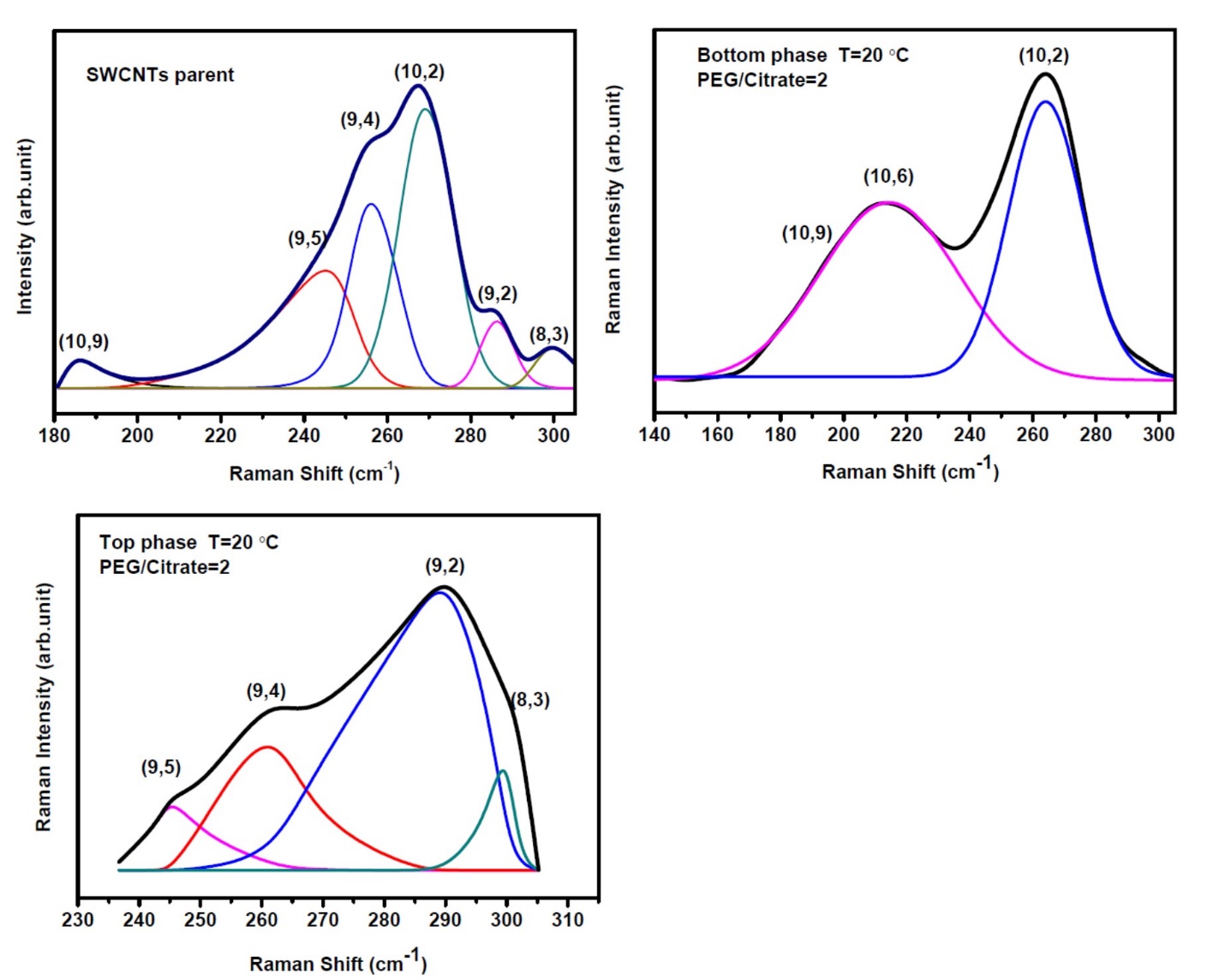


Figure S3. The resonance Raman spectroscopy (RBM mode-diameter relation) taken from starting material, top phase, and bottom phase composition of PEG: citrate salt with ratio 2, (F1).
